# Six-fold over-representation of graduates from prestigious universities does not necessitate unmeritocratic selection in the faculty hiring process

**DOI:** 10.1371/journal.pone.0185900

**Published:** 2017-10-04

**Authors:** Michael Miuccio, Ka-yuet Liu, Hakwan Lau, Megan A. K. Peters

**Affiliations:** 1 Department of Psychology, University of California Los Angeles, Los Angeles, California, United States of America; 2 Department of Sociology, University of California Los Angeles, Los Angeles, California, United States of America; 3 California Center of Population Research, University of California Los Angeles, Los Angeles, California, United States of America; 4 Brain Research Institute, University of California Los Angeles, Los Angeles, California, United States of America; 5 Department of Psychology, University of Hong Kong, Hong Kong; 6 Department of Bioengineering, University of California Riverside, Riverside, California, United States of America; University of Oxford, UNITED KINGDOM

## Abstract

To achieve faculty status, graduating doctoral students have to substantially outperform their peers, given the competitive nature of the academic job market. In an ideal, meritocratic world, factors such as prestige of degree-granting university ought not to overly influence hiring decisions. However, it has recently been reported that top-ranked universities produced about 2–6 times more faculty than did universities that were ranked lower [1], which the authors claim suggests the use of un-meritocratic factors in the hiring process: how could students from top-ranked universities be six times more productive than their peers from lower-ranked universities? Here we present a signal detection model, supported by computer simulation and simple proof-of-concept example data from psychology departments in the U.S., to demonstrate that substantially higher rates of faculty production need not require substantially (and unrealistically) higher levels of student productivity. Instead, a high hiring threshold due to keen competition is sufficient to cause small differences in average student productivity between universities to result in manifold differences in placement rates. Under this framework, the previously reported results are compatible with a purely meritocratic system. Whereas these results do not necessarily mean that the actual faculty hiring market is purely meritocratic, they highlight the difficulty in empirically demonstrating that it is not so.

## Introduction

Is academia a pure meritocracy? If it is not, what makes it deviate from the ideal? Doctoral students now seem to have to substantially outperform their peers in the competitive academic job market to get a faculty position, and the prestige of the degree-granting institution appears to be a crucial factor. A recent study found that top-ranked universities produced about 2–6 times more faculty than did universities that were ranked lower [1]. A multitude of mechanisms, including non-meritocratic factors, have been suggested to underlie the differences in placement rates across institutions of varying status, e.g., nepotism, racism, and sexism, and, at the institutional level, hiring network structures and prestige of the programs [1–6].

While these previous studies provide good evidence that the academic market may not be a pure-meritocracy, the role of the increasingly high level of competition of academic job markets in generating the observed uneven distribution of jobs as a function of institutional prestige is less well discussed. It is reasonable to expect the qualifications of successful candidates to increase with the level of competition in the job market. That said, one might question whether students from top-ranked universities could outperform their peers by as much as six times in productivity to justify the six-fold difference in placement rates [1]. (Certainly, it has been pointed out that the mapping of success and quality (however it is measured) does not have to be linear *even* in a purely meritocratic system. Curvilinear relationships between productivity and return are not unique to the faculty hiring markets, and have been referred as the “superstar” [7] and “winner-take-all” effects [8].)

Our contribution to this broader literature is that we use a simple mathematical model of binary decisions to quantify the relationship between the competitiveness of the market and the return rates to productivity. Using faculty hiring as an example, we show that a signal detection theoretic argument is consistent with the large discrepancies in faculty production while supposing largely similar rates of productivity between top-ranked and lower-ranked universities.

Signal detection theory [9,10] was developed in World War II to study the detection of information bearing signal in radar when there is noise in the system. Psychologists have also picked up it as a mathematical model to quantify how humans make binary decisions when there is uncertainty. One of the key insights, regardless of the system or observer, is that the criterion that is being used to make the decision is as important as the information in the signal itself.

According to signal detection theory, a decision maker uses a criterion to set a threshold, classifying evidence values above that threshold as belonging to one category, and values that fall below it as belonging to the other category. Setting a high criterion can result in large differences in the *proportion* of evidence that surpasses the threshold for two seemingly similar distributions. Let us take a simple example to see how this might occur. If you are in the shower, you will hear the noise of the water falling from one moment to the next. Imagine you are also trying to listen for a phone call, so you want to discriminate moments when you hear the “pure noise” of the shower versus the “noise plus signal” of the phone ringing audibly over the noise of the shower. In this classic example, perhaps the phone call is not very important, so you set a high criterion for deciding that you hear the phone ringing, which would prompt you to hop out and answer it.

**Fig 1:**
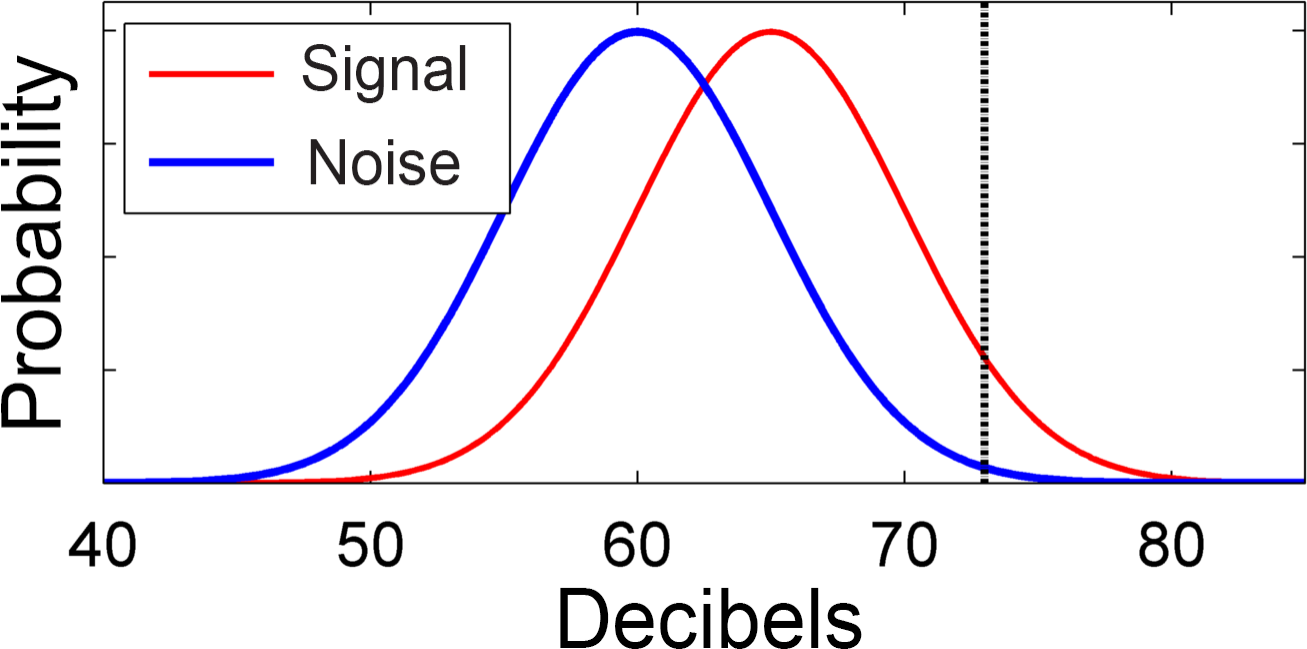
Illustration of the effect of an extreme criterion on categorization according to signal detection theory. Shown is an arbitrary, imaginary example of distributions of the noisy evidence an observer might have access to based on a “pure noise” source versus a “noise plus signal” source. For example, if you are in the shower, at any given moment you will hear the noise of the water falling; if, however, you are trying to listen for a phone call at the same time, you will want to try to discriminate cases where what you hear is due to the “pure noise” of the shower, or the “noise plus signal” of the phone ringing audibly over the noise of the shower. In this arbitrary example, you are enjoying your shower and are not inclined to hop out at the slightest provocation, so you set a very high threshold, or criterion, for deciding that you hear the phone ringing on the basis of what you hear. The distributions of noisy evidence differ in mean between signal and noise by only 5 decibels, but if the criterion for deciding that what you hear is “signal” is set to an extremely high value, such as 73 decibels (“irritatingly loud”), the chance that you will jump out of the shower because the phone is *actually* ringing (the area above the criterion under the red curve) will be *many* times (in fact, 10.8 times) more than the chance that you will jump out of the shower when it is *not* ringing (the area above the criterion under the blue curve).

Fig 1 shows example distributions representing the loudness of “noise plus signal” (i.e., shower noise plus the phone is ringing; red) and “pure noise” (i.e., just the shower; blue), with signal on average 5 decibels louder than pure noise (standard deviations are arbitrarily set to 5 decibels for both distributions for the purposes of this illustrative example). Let’s suppose that if you hear a noise that is above 73 decibels (“irritatingly loud”), you classify it as “phone is ringing”. With this high criterion set, it becomes clear that the *proportion* of the time you will get out of the shower when the phone is *actually* ringing (i.e., noise plus signal; the area above the criterion under the red curve) is over ten times larger than the *proportion* of the time you will hop out when it is *not* ringing (the area above the criterion under the blue curve), despite the fact that the distributions’ means differ only by a few decibels. A lower criterion (e.g., you will get out of the shower if you hear a sound 60 decibels or more) would not produce such an extreme asymmetric effect, with the proportion of “I think it’s ringing” cases when it is *actually* ringing being only 1.6 times the proportion of “I think it’s ringing” cases when it is *not*.

We formalized this theoretical argument as a signal detection model, to be applied to the faculty job market by utilizing the distributions of meritocratic measures of productivity that are likely to exist in true doctoral student populations. We then demonstrated the validity of the theoretical argument by collecting a small sample of graduate students from selected psychology departments in the United States as a proof-of-concept exercise.

Supporting the conditions of the signal detection theoretic model, we found that the productivity of graduate students in the high- and low-tier universities in our sample is actually very similar. However, also in support of the model, faculty production appeared disproportionately skewed toward higher-tier schools. While we cannot rule out the roles of other factors in faculty hiring decisions, such as institutional prestige or social networks as has been previously suggested, the result is also consistent with a high set criterion for faculty hiring. In other words, whereas the model—and these sample data—do not necessarily mean that the actual faculty hiring market is purely meritocratic, they highlight the important role of an extreme hiring criterion due to a keenly competitive environment. They also reveal the difficulty in empirically showing that the faculty job market is demonstrably “corrupted” by un-meritocratic factors.

## Materials, methods, and results

### Signal detection theoretic model

Our signal detection theoretic model proceeds much as the above example of listening for a phone call while in the shower. In signal detection theory, the *samples* of data available to a decision maker are typically assumed to have been drawn from distributions with known shape and parameters [9,10] (although the argument also holds for nonparametric probability distributions). For the purposes of examining faculty hiring rates, we assume that these samples represent some form of meritocratic productivity scores given to individual students, such that students from higher ranking institutions constitute a distribution of Higher Tier productivity scores and those from lower ranking institutions constitute a distribution of Lower Tier productivity scores. In our model, we will refer to these productivity score samples for individuals as *x*, i.e. samples of the random variables *X*_*Higher tier*_ and *X*_*Lower tier*_. Because these meritocratic scores cannot be negative and are positively skewed, we can assume the known shape of these random variables to be exponential, such that for all students *f*(*x*) ∝ *λe*^−*λx*^. (See Proof-Of-Concept Example, below, for validation of these assumptions, and additional demonstration that the validity of the model’s predictions does not rest on these assumptions.) The proportionality occurs because the distribution is normalized such that they constitute probability density functions.

Following the shower example above, if a student has a productivity score *x* that falls above an acceptable value—i.e., a hiring criterion—then he or she will be hired as a faculty member. If a student’s score *x* does not exceed the hiring criterion, he or she will not be hired. (Of course, the hiring criterion is a soft criterion, in that students who do not meet or exceed an arbitrary cutoff may still be hired as faculty. To simplify the initial theoretical exercise, we assume the criterion to be a hard boundary; we later relax this assumption and show the argument still holds.)

To determine the magnitude of this hiring criterion, we first must determine the probability of being hired as faculty. A simplistic formula for this probability of being hired, *p(hire)*, would be to compare the average number of students who graduate from each per year to the average number of faculty hires made at those same universities per year. Thus, we can define for a given year

$$p(hire)=\frac{\#\;faculty\;hires}{\#\;graduates}$$ (1)

The hiring criterion is defined as the meritocratic productivity score at which the area above this criterion under the distribution of *all* students’ productivity scores regardless of university ranking, *f*(*x*), matches *p(hire)*. Thus, mathematically the criterion *c* is defined as

$$p(hire)=H(c)=1-F(c,x)=1-\int_{0}^{c}f(x)dx=\int_{c}^{\infty }f(x)dx$$ (2)

leading to

$$c=H^{-1}(p(hire))$$ (3)

where *H(c)* defines the function giving *p(hire)*, which is inverted in Eq 3, and the integral initially is taken from 0 to *c* because an exponential function is undefined at *x* < 0.

To evaluate how the criterion will differentially affect the conditional probability of being hired as faculty depending on degree-granting institution, *p(hire|university tier)*, we examine the proportion of each conditional probability density function for each university ranking tier that falls above the criterion *c*. Conditioned by the rank of the student’s training institution, the distribution of meritocratic productivity scores is $f(x|tier)\propto \lambda_{tier}e^{-\lambda_{tier}x}$, where *λ*_*tier*_ refers to the fitted *λ* parameter for each university tier, and thus the conditional probability of being hired is defined as

$$p(hire|tier)=H(c|tier)=\int_{c}^{\infty }f(x|tier)dx$$ (4)

It is assumed that *p(hire)* is quite low due to the competitiveness of the academic job market, with under 10% of graduates with doctoral degrees being hired as faculty members [11] (see also our Proof-of-Concept Example for validation). At this low *p(hire)* the criterion for being hired will be quite high, as a small proportion of the distribution of all meritocratic productivity scores ought to fall above the criterion. Under a high criterion, modest differences in students’ productivity between universities tiers (quantified as differences in parameter values *λ*_*tier*_) can lead to radically imbalanced areas under Higher and Lower tier functions above the hiring criterion, *p*(*hire|tier*)—just as with modest mean decibel differences between pure noise and signal plus noise in the shower example in the Introduction. And, as with the shower example, a less extreme criterion leads to less asymmetry in hiring rates.

Thus, the signal detection theoretic model predicts the following: (a) students from Higher and Lower tier universities will have similar levels of productivity; (b) under an extremely competitive academic job market with a very high hiring criterion, small differences in student productivity across university tier will lead to manifold differences in probability of being hired; and (c) under a less competitive job market, when the hiring criterion is less extreme, these asymmetries will be reduced.

### Proof-of-concept example

#### Data collection

Rather than demonstrate the predictions of the signal detection theoretic model with arbitrary simulated data, we elected to demonstrate a more illustrative proof of concept by using a realistic sample of Psychology students’ productivity across universities of different rankings on the 2016 *U*.*S*. *News & World Report* list. This discipline was chosen because Psychology has little ‘leakage’, i.e. there are minimal differences between the department discipline an individual graduates from and the department discipline in which he or she is ultimately hired. Psychology also has the additional benefit of an easily-defined (albeit simplified) objective meritocratic measure based on the impact factors of peer-reviewed publications for each individual.

From universities listed on the National Universities Rankings from the *U*.*S*. *News & World Report*, we collected three samples of individuals in their Psychology departments depending on university rank—rank 1–10, rank 11–20, and rank 21–100—through a combination of online search of student directories and direct contact with departments. Overall, we collected data on 1871 individuals from 26 institutions.

#### Defining a meritocratic measure

In signal detection theory, each discrete sample (in the shower example, what you hear at each ‘moment’) represents a draw from a probability distribution around a true mean but corrupted by noise. For our meritocratic productivity score we defined the simple metric of Impact Factor Sum (IFS) for each individual in this sample as the sum of the impact factors for every publication that individual had authored regardless of authorship order, as indexed in Google Scholar. This method was used as a means to equitably search all students’ publications because not all students post their CVs, and data downloaded from large archives (e.g., PubMed) would by definition exclude individuals with no publications and journals not indexed by that engine. We also wanted to quantify productivity for all current students, not graduates. Importantly, this definition means that IFS is completely independent of the university rankings from the *U*.*S*. *News & World Report* used to define university rank.

Although other more complete and complex indices are available—e.g., h-index [12,13] or predictions based on machine learning techniques [14] —we elected to use this IFS metric due to its simplicity and close relationship with more complex metrics. Specifically, it has been shown that the perceived quality (i.e., impact factor) of a publication is given more weight in the faculty hiring process than its actual quality (i.e., its citation rate), and that the two most important factors in predicting faculty hiring are impact factor of publications and number of publications [11]; we therefore combined these factors into a single IFS. Although this simplified IFS metric does not cover all possible facets of meritocratic success, we remind the reader that these data are intended to provide a proof of concept illustration of our signal detection theoretic model.

#### Defining university tiers

IFS for each individual in our entire sample ranged from 0 (no publications) to 304.637 (many first-, middle-, and last-authorship papers in high-impact journals) (μ = 7.530, σ = 19.403). These were collected across three groups corresponding to the tier of the university (according its *U*.*S*. *News & World Report* ranking) from which an individual had received his or her doctorate: rank 1–10 (n = 607, range: 0–304.637, μ = 11.655, σ = 26.929), rank 11–20 (n = 532, range: 0–96.17, μ = 5.751, σ = 13.332), and rank 21–100 (n = 732, range: 0–150.87, μ = 5.629, σ = 14.846). Importantly, because IFS is based on Google Scholar profiles but university tier is based on rankings from the *U*.*S*. *News & World Report*, IFS should not be based *a priori* on university rank if there is no underlying relationship between the two.

Although typically Gaussian distributions are used in signal detection theory, this is because noise is assumed to be normally distributed; it is not necessary that distributions be Gaussian when empirically it can be demonstrated that they are not, as is the case here. Here, we observed that IFS was approximately exponentially distributed for all individuals and in each university tier, because by definition IFS ≥ 0 and is positively skewed (see below for additional explorations that do not assume exponential distributions). Because IFS in each tier is not normally distributed, we used nonparametric tests to compare them. We tested for differences among the three samples (rank 1–10, 11–20, and 21–100) with (a) Wilcoxon Rank-Sum (Mann-Whitney U) tests, which are nonparametric tests that do not rely on assumptions of normality (Wilcoxon, 1945), and (b) Kolmogorov-Smirnov nonparametric tests, which test for differences between probability distributions and are sensitive to both mean and distribution shape [15]. Wilcoxon Rank-Sum (Mann-Whitney U) tests revealed significant differences in IFS between universities with rank 1–10 and those with rank 11–20 and 21‒100, but no differences between universities with rank 11–20 and 21–100 (Table 1, top three rows). Kolmorogov-Smirnov tests revealed an identical pattern (Table 1, top three rows).

**Table 1:** Results of nonparametric comparisons of all university tier groups.

				Wilcoxon Rank Sum / Mann-Whitney U		Kolmorov-Smirnov	
	Rank pairing	n_1_	n_2_	U	p	D	p
**Individual groups**	1–10 vs. 11–20	607	532	3.641e5	< .001*	0.134	< .001*
	1–10 vs. 21–100	607	732	4.368e5	< .001*	0.156	< .001*
	11–20 vs. 21–100	532	732	3.420e5	.319	0.054	.318
**Merged groups**	Higher (1–10) vs. Lower (>10)	607	1264	6.163e5	< .001*	0.146	< .001*

All individual groups’ pairwise comparisons are significant (denoted with *) except for rank 11–20 vs. 21–100 (top three rows). We therefore collapsed across the two similar lower groups to create a single pair of groups (bottom row). This pair of groups—Higher vs. Lower tier—was used in all further analyses.

Because these tests revealed indistinguishable IFS distributions for universities with ranks 11–20 and 21–100, we collapsed across the two lower-ranking groups to create a Higher tier sample (n = 607, rank 1–10) and a Lower tier sample (n = 1264, rank >10). The range of IFS scores for the Higher tier was therefore 0–304.637 (μ = 11.655, σ = 26.929), and for the Lower tier was 0–150.87 (μ = 5.680, σ = 14.223). These two tiers are significantly different from each other (Table 1, bottom row).

#### Comparing university tiers on meritocratic measures

We evaluated the similarity between the remaining Higher tier (rank 1–10) and Lower tier (rank >10) groups using Receiver Operating Characteristic (ROC) analysis, which plots the *hit rate* versus *false alarm rate* at varying criterion values [9,10]: for all possible criterion values, IFS values are defined as *hits* if they are correctly classified as belonging to the Higher tier group, and *false alarms* if they are classified as belonging to the Higher tier group but actually came from the Lower tier group. At each possible criterion value the *hit rate* and *false alarm rate* are calculated, which are then plotted against each other to form the Receiver Operating Characteristic (ROC) curve. The area under this ROC curve (AUC) is a measure of the similarity between the Higher and Lower tier IFS scores, as it provides a normalized metric of separability of distributions: AUC = 0.5 indicates distributions are identical, and AUC = 1 indicates distributions are completely separable. This nonparametric comparison also removes reliance on any assumptions of normality in the distributions to be compared, and indeed removes reliance on the distributions being parametric at all. Although signal detection theoretic investigations of psychological phenomena are often assumed to rely on normally-distributed samples around a true environmental mean for the sake of simplicity [10], nonparametric signal detection theoretic analyses do not rely on this assumption.

**Fig 2:**
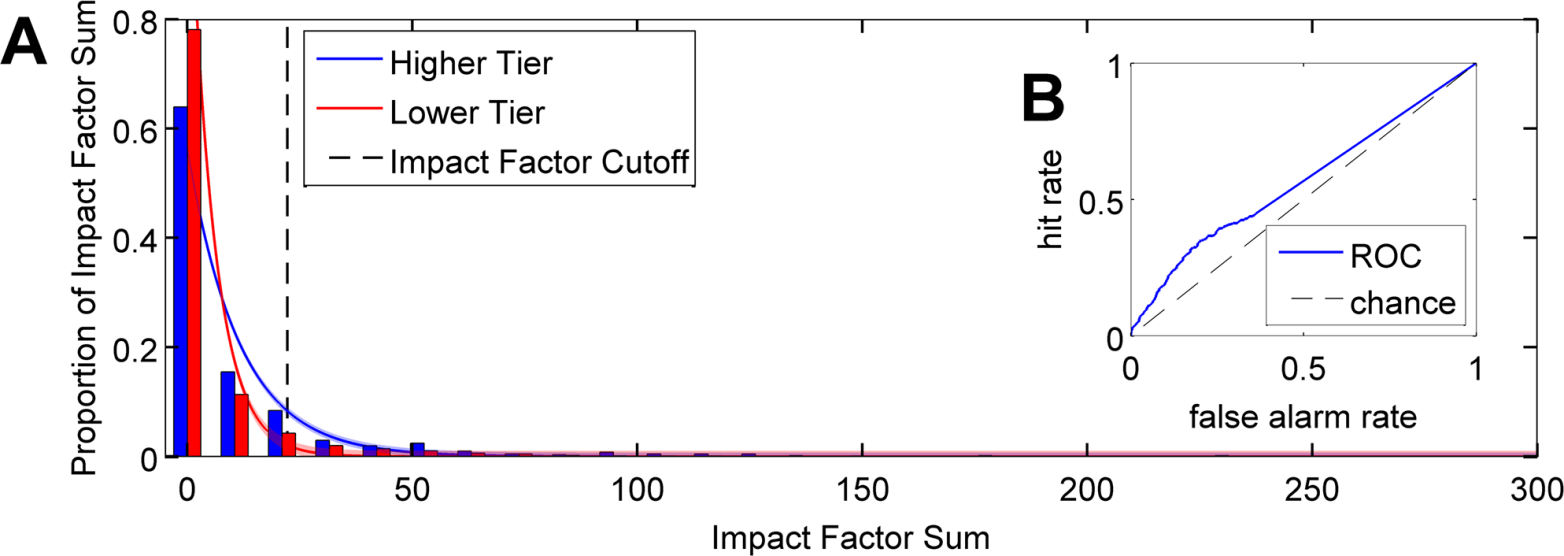
Distributions of Impact Factor Sum (IFS) across university tier are very similar. Panel (a) shows that productivity (graduate students’ IFSs) of the different university tiers (Higher vs. Lower) is quite similar while the criterion for getting a faculty position is, as expected, fairly high. The difference between the two IFS distributions for Higher and Lower tiers is minimal, as shown by the ROC curve in (b): the area under the curve (AUC), representing discriminability between Higher and Lower tier universities, is 0.563. This is almost at chance (chance AUC = 0.50), showing that the distributions are nearly equivalent, mimicking the 5-decibel loudness difference between noise and signal plus noise in our simple shower example in the Introduction. To calculate the location of the IFS criterion to be hired as faculty (i.e., IFS Cutoff), we fitted an exponential function to the overall distribution of IFS, which represents productivity of all graduate students regardless of university (fitted curve not shown). The IFS Cutoff was calculated to be 22.20 in accordance with the reality of faculty production. The percentage of the area under the two curves representing the Higher and Lower tiers that falls above this IFS Cutoff, *i*.*e*., the probability of a graduate student getting a faculty position after graduating from *any* university, is about 5%.

Despite significant differences in IFS distribution between the Higher and Lower tiers (recall that tiers are defined by the *U*.*S*. *News & World Report* and not by IFS score) at the statistical level, the samples appear visually similar (Fig 2A), and ROC analysis [9,10] showed that the normalized magnitude of this difference was indeed quite slight, at AUC = 0.563 (Fig 2B). The interpretation of this AUC value is that if a random person is picked from either cohort and you are asked to guess the cohort to which the person belongs based on his or her IFS score, your likelihood of being correct would be just 6.3% above chance (chance = 50%, confirmed with permutation tests). This similarity between the two cohorts is also reflected by the median score for both distributions: both Higher and Lower tier groups have a median IFS of 0. So the distributions’ differences are highlighted primarily in the extreme end of their upper tails, mimicking the 5-decibel loudness difference between noise and signal in the example in the Introduction and demonstrating the first model prediction. All IFS data are available in S1 File (available online).

#### Estimating the probability of being hired as faculty

To define the criterion in IFS space for being hired as faculty regardless of graduate university, as described in the Model section above, we estimated the probability of being hired as faculty across a large number of universities. To do this, we collected a fourth sample from the Psychology departments across all *U*.*S*. *News and World Report* ranks from 1–50, to compare the average number of students who graduate from each per year to the average number of faculty hires made at those same universities per year.

In this sample of graduates and hires from Psychology departments at 22 institutions, we found that an average of 682.6 students graduated per year, and an average of 35.5 individuals were hired as faculty at those same institutions per year. By Eq 1, this leads to *p(hire)* = 0.0520, meaning that approximately 5% of all graduates with doctorates in Psychology are hired as faculty in any given year. This result is in line with previous reports of annual faculty hiring rates of about 6.2% [11].

#### Setting the criterion for being hired as faculty

To calculate a criterion for being hired within the overall IFS distribution, we collapsed all IFS for all individuals in the Higher and Lower tier groups regardless of university tier. We then used bootstrapping to ensure that our demonstration of the model’s predictions was not overly sensitive to our particular sample. Each ‘loop’ of the bootstrapping procedure can be thought of as a given year’s searches for one or more faculty members: for each bootstrapped set of ‘searches’, a random sample of 1000 IFS data points (with replacement) was drawn from this overall IFS distribution, mimicking the distribution of individuals who might apply for the current year’s job postings. To each sample of 1000 ‘applicants’, we fitted an exponential function of the form *f*(*x*) ∝ *λe*^−*λx*^ to all individuals’ IFS scores regardless of university tier, which we then normalized so that it would constitute a probability density function over the range of IFS in the current year’s ‘applicant pool’ (see Signal Detection Theoretic Model, above). We then calculated the criterion *c*, or IFS Cutoff, according to Eqs 2 and 3, which implies that the top ~5% of applicants would be hired in any given year. This process was repeated 1000 times for a total of 10,000,000 samples, leading to 1000 estimates of *c*. We found mean *c* = 22.20 (median = 22.20, σ = 1.74) (Fig 2), meaning that, if we live in a meritocracy, any given individual should aim to have a total IFS equal to or exceeding 22.20 if he or she hopes to be hired as a faculty member in a particular job cycle.

#### Extremely unequal probability of being hired as a function of university tier

**Fig 3:**
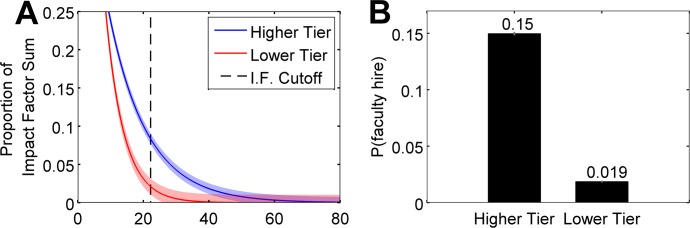
Zoomed-in view of IFS distributions and probability of faculty hire conditioned on university tier, *p(hire|tier)*. Panel (a) shows the portion of the graph from Fig 2A nearest the criterion. At this zoom level it is clear that despite their overall similarity, the distributions are quite different at the relatively extreme value of the IFS criterion. Shaded regions indicate the standard error of the mean (SEM) across bootstrapping loops (see Methods) at each IFS value. Panel (b) shows the average probability of being hired as faculty conditioned on having graduated from a Lower or Higher tier university, or mean *p(hire|tier)* across bootstrapped samples (see Methods). According to our sample, the mean probability of being hired after graduating from a Higher tier university is 14.98%, or about eight times the mean probability of being hired as faculty after graduating from a Lower tier university (1.88%). Error bars represent the SEM across all bootstrapping loops.

Despite the visual similarity between the Lower and Higher tier distributions of IFS (Fig 2A), closer inspection of the tail ends of the distributions, above the criterion *c*, reveals important differences. Fig 3A displays the mean of *f(x|tier)* for both tiers over all loops of the bootstrapping analysis zoomed in on the region of the IFS Cutoff criterion *c*, with SEM across bootstrap loops represented by the shaded regions. The Higher tier IFS scores display a strikingly large advantage over the Lower tier IFS scores at the location of the criterion.

To evaluate how the high criterion might lead to potentially exaggerated differences if we conditioned on university tier, i.e. *p(hire|tier)*, we again used bootstrapping to ensure that our results were not overly sensitive to our particular sample. As before, on each bootstrapped ‘job cycle’, a random sample of 1000 IFS data points (with replacement) was drawn from each of the Higher and Lower tiers, respectively, and an exponential function fit to the sample of the form $f(x|tier)\propto \lambda_{tier}e^{-\lambda_{tier}x}$, where *λ*_*tier*_ refers to the fitted *λ* parameter for each university tier. Following normalization such that the fitted functions constituted probability density functions, we used Eq 4 on each bootstrapped ‘job cycle’ for each tier to calculate the probability of being hired conditioned on university tier. This process was repeated 1000 times for a total of 20,000,000 samples (10,000,000 from each of the Higher and Lower tiers), leading to 1000 estimates of *p(hire|Higher tier)* and 1000 estimates of *p(hire|Lower tier)*.

Fig 3B displays the mean and standard error of *p(hire|tier)* for the Higher and Lower tier universities across all loops of the bootstrapping analysis. Graduates of Higher tier universities are significantly more likely to be hired as faculty (t(999) = 144.839, p < .001), and by nearly a factor of eight: you are almost eight times as likely to be hired as faculty if you receive your doctorate in Psychology from a top-10 university than if you attended any university of lower rank, based purely on the simplified, meritocratic metric of IFS. This occurs despite the high degree of similarity in the IFS distributions for Higher and Lower tier universities (AUC = 0.563), as a result of small but significant differences in the probability densities of these distributions at and above the high hiring criterion. This demonstrates the second model prediction.

#### A ‘softer’ hiring threshold?

One might be concerned that the hard cutoff at *c* = 22.20 is unrealistic, because not all applicants with IFS ≥ 22.20 are hired, nor are all applicants with IFS < 22.20 rejected. We therefore repeated the above analysis with an additional noise term added, such that c* = c + ε, with *ε* ~ *N*(*μ* = 0, *σ* = 10). We arbitrarily selected *σ* = 10 to allow for a large amount of ‘softness’ or ‘subjectivity’ in each individual ‘job search’. However, the results were the same for all reasonable values for *σ* that we tested: these simulations revealed that even with a ‘softer’ threshold from one ‘job cycle’ or ‘job search’ to the next, if *on average* the criterion is extreme (because of the competitive job market), the asymmetry in hiring rates between Higher and Lower university tiers is nearly identical to the fixed criterion version of the simulation (t(999) = 138.838, p < .001).

#### Asymmetry in hiring rates is a direct consequence of an extreme criterion

An important lesson from our signal detection theoretic model is that the extreme criterion (i.e., low probability of being hired as faculty) set by a keenly competitive faculty job market is largely responsible for the large asymmetry in faculty hiring rates. The effect of the extreme criterion is clear in our proof-of-concept example dataset, the criterion for being hired at IFS ≈ 22 reflects the externally valid hiring rate of ~5–6% [11], and leads to hiring asymmetry by a factor of nearly eight between Higher and Lower tier universities.

**Fig 4:**
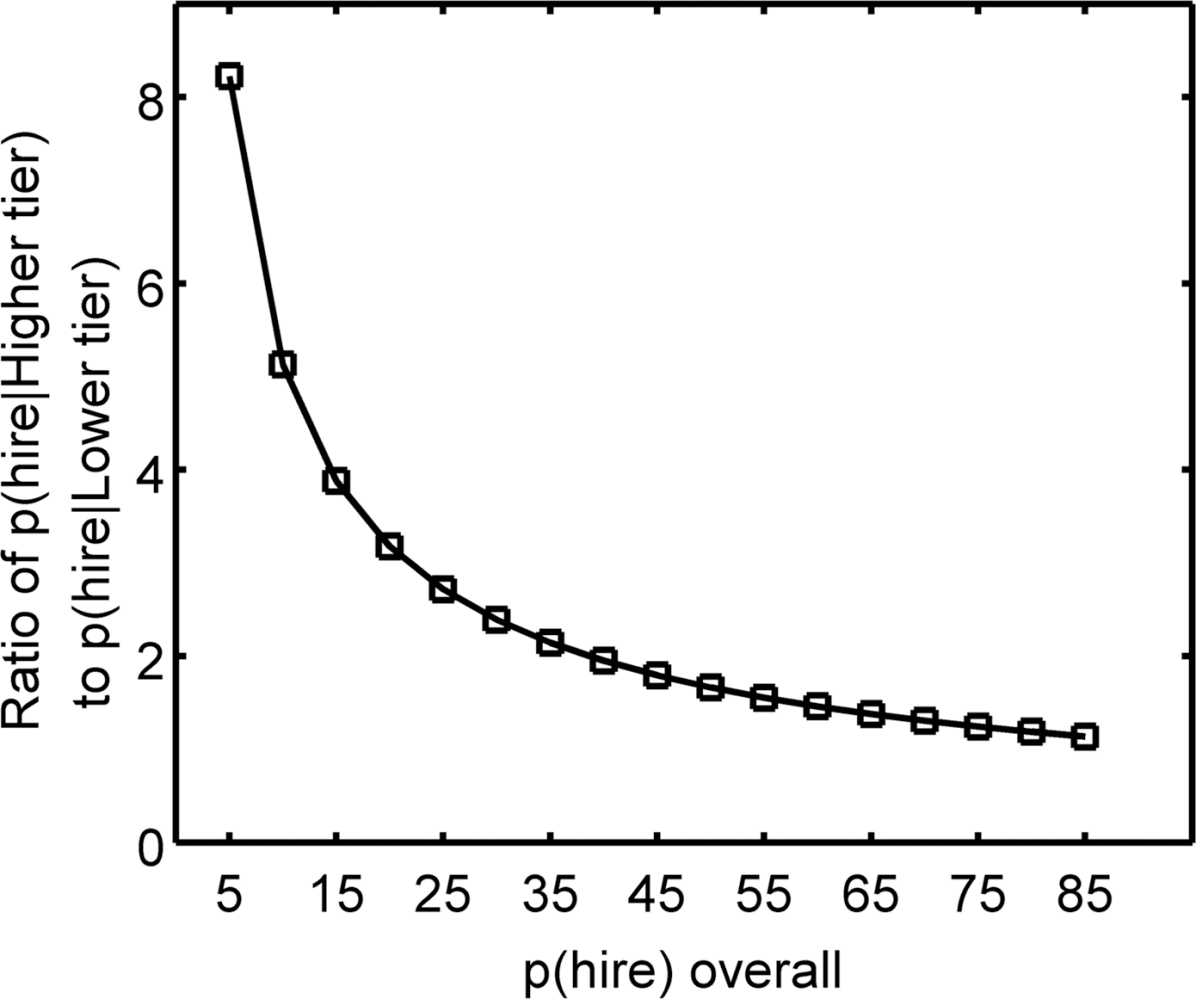
Less extreme hiring criterion values lead to less pronounced hiring asymmetry between university tiers. By shifting the criterion to more liberal values—from *p(hire)* = 0.05 to *p(hire)* = 0.85—we show that the hiring asymmetry is reduced and ultimately disappears almost entirely. The appearance of a non-meritocratic system is thus perpetuated by the severe competitiveness of the current hiring climate.

If the hiring climate were less competitive, with a less extreme hiring criterion along any meritocratic dimension, this asymmetry would dwindle with increasing values for *p(hire)* and eventually disappear (Fig 4). To illustrate this consequence of the signal detection theoretic model, we calculated the hiring rate asymmetry between Higher and Lower tier universities (Eqs 3 & 4) as a function of increasing *p(hire)*, i.e. decreasing competitiveness of the academic job market. As above, we also used bootstrapping analysis with 1000 samples of IFS scores from the overall distribution. Although the mean *p(hire)* ratio between Higher and Lower tier universities starts high, as would be expected in the current competitive hiring climate, it dwindles with decreasing competitiveness, asymptoting as *p(hire)* approaches 1. This demonstrates the third model prediction. Thus, the *appearance* of a non-meritocratic system is in fact perpetuated in large part by the extreme difficulty of attaining a faculty position.

#### Effect of focusing on more advanced students

To demonstrate that the validity of our signal detection theoretic model is not dependent on the idiosyncrasies of any particular sample, we repeated the above-described analyses excluding individuals with IFS = 0 from both Higher tier and Lower tier samples (n_Higher_ = 272, n_Lower_ = 463). This can be thought of as selecting primarily the more advanced students while removing first- and second-year students who have not published yet, to alleviate concerns that the proof-of-concept results reflect the relatively large proportion of students who had no publications at the time of our data sample collection.

Analyses on this subsample of individuals shows no change in overall findings. Firstly, higher tier universities still stand out from universities with lower rankings in terms of student productivity (Table 2), justifying the collapsing of the lower two university tiers. Further, just as in the main analysis, despite a higher IFS Cutoff (criterion) at 57.05, resulting from the shift of probability density towards higher IFS values, the observed similarity between Higher and Lower tier distributions is maintained (albeit a little lower, AUC = 0.633), and resultant ratios of Higher to Lower tier faculty hiring rates are similarly starkly asymmetric: mean *p(hire|Higher tier)* = .108, mean *p(hire|Lower tier)* = .025. This result demonstrates that while our sample may be small and IFS Score may not capture all possible meritocratic elements, the proof of concept of our signal detection theoretic argument does not depend on any specific sample.

**Table 2:** Results of nonparametric comparisons of Higher and Lower tier groups after individuals with IFS = 0 have been removed.

				Wilcoxon Rank Sum / Mann-Whitney U		Kolmorov-Smirnov	
	Rank pairing	n_1_	n_2_	U	p	D	p
**Individual groups**	1–10 vs. 11–20	272	201	7.118e5	< .001*	0.211	< .001*
	1–10 vs. 21–100	272	262	8.281e5	< .001*	0.251	< .001*
	11–20 vs. 21–100	201	262	4.827e5	.250	0.104	.162
**Merged groups**	Higher (1–10) vs. Lower (>10)	272	463	1.169e5	< .001*	0.232	< .001*

#### Concerns about distribution shape

It is true that the distributions of IFS used here in our proof of concept example deviate from the traditional distributions used in signal detection theoretic arguments. The traditional approach—as in our shower and phone example—is to use Gaussian distributions which represent noisy samples around a true mean, with normally-distributed noise. We note that in the present study, the IFS score for a particular individual likewise represents a noisy point estimate of the “true” productivity or ability for a given group of individuals. For example, there are plenty of cases where very good institutions produce students who are not very productive, or where lower-ranked institutions produce superstars. Yet it is also true that the use of exponential distributions of IFS (and its resultant minimum at IFS = 0) instead of Gaussian distributions does not substantially impact the signal detection theoretic argument demonstrated here. To confirm this, we re-ran the above analyses using ‘more advanced’ students using a log-transform of IFS, without the IFS = 0 scores (because log(0) is undefined). While these distributions of IFS for Higher and Lower tier universities appeared approximately normal, the distribution of IFS for the Higher tier was confirmed to be Gaussian with a Lilliefors test [16] but the distribution for the Lower tier failed this test of normality. Therefore, we opted to use kernel density estimation to approximate the nonparametric probability density functions of log(IFS) for both Higher and Lower tier universities, and re-run the bootstrapping analysis. Importantly, this approach does not require that a minimum of log(IFS) be dictated by the parametric nature of any particular distribution, or that the probability distribution of IFS take on any particular shape at all.

This approach revealed the same pattern of results as reported above. Mean log(IFS) for the Higher tier was only slightly above that for the Lower tier, at μ = 2.633 and μ = 2.107, respectively. Yet with a high criterion, calculated to be on average *c =* 4.317 in natural log space, the hiring rate asymmetry is still many times the difference in means, with *p(hire|Higher tier)* = .082 and *p(hire|Lower tier)* = .033. These results demonstrate that, except in scenarios that are *a priori* highly unlikely in the real world (e.g., when the probability distributions in question are uniform, or when distributions significantly deviate from each other in shape), the signal detection theoretic argument here will hold regardless of the exact shape or family of probability distributions used.

## Discussion

Here, we have used a simple signal detection model [9,10] to demonstrate how surprisingly extreme asymmetries in faculty hiring rates for graduates of Lower and Higher tier universities need not necessitate non-meritocratic factors in the faculty hiring process. Importantly, the theoretical model does not depend on particularities of distribution shape, distribution parameters, or sample size. (The exception to this is when the probability distributions in question are *a priori* highly unlikely in the real world, such as uniform distributions which would lead the difference in *p(hire|tier)* to be linearly related to the difference in means between the two distributions, or some cases where two distributions have qualitatively different shapes.) Simply put, as long as the faculty job market is competitive—i.e., a high meritocratic criterion exists because the probability of being hired is low—small differences in productivity as a function of university rank can easily be magnified into manifold differences in faculty production rates. While it has been shown that doctoral prestige better predicts faculty placement than productivity [2] (but see [11,17]), our theoretical argument casts doubt on the supposition that extreme hiring asymmetries *must* imply either vast (and unrealistic) differences in productivity, or a strongly non-meritocratic system.

We believe that unmeasured heterogeneities in the positions or in the candidates are certainly important in the hiring decision. That said, our model provides an alternative explanation to why controlling for publication records usually has only modest effects on the prestige-placement relationship (e.g., [18]). More importantly, our study shows that, in principle, such small differences can aggregate into large differences in the placement records across institutions, even in a pure-meritocracy.

That decision-makers likely consider factors beyond Impact Factor Scores in the selection process may explain why the observed differences in placement across institutions are less than what our model predicts. It may also explain the heterogeneity we observed in actual faculty hiring decisions. However, what our modeling exercise demonstrates is the difficulty in quantifying the importance of unobserved factors when the market becomes extremely competitive.

### Proof-of-concept example

We used a realistic sample of data to demonstrate the consequences of our signal detection theoretic model, which revealed that true rates of faculty production are highly skewed towards Higher tier universities by a factor of eight even though student productivity between tiers is nearly indistinguishable (AUC = 0.563). The high criterion (IFS Cutoff) in our sample (dictated by an extremely competitive faculty hiring system) is reflective of actual faculty hiring rates both in our sample and as reported by others (5–6% [11]), thus providing a realistic dataset with which to demonstrate the predictions of our signal detection theoretic model. We also showed that this asymmetry will dissipate only if the hiring criterion becomes less extreme, reaching a factor of 2 (Higher tier graduates have double the chance of being hired compared to Lower tier graduates) when the overall probability of being hired reaches ~35% (Fig 4) and becoming almost equal when *p(hire)* reaches ~85% or higher.

Our example has several limitations. One possible limitation is the means by which we gathered data to calculate IFS based on Google Scholar publication results. We elected to collect publication data via Google Scholar because (a) individuals do not have control over what appears in the search engine (unlike their appearance in NeuroTree or uploaded CVs) and (b) we wanted a metric by which to quantify all students’ productivity as a function of university tier including students who had published nothing at all. By searching for individual students’ names (collected from student rosters) on Google Scholar we were able to calculate all students’ IFS scores for all papers they had published, if any. This helped to keep our sampling method equivalent among all the universities we sampled from.

Another possible concern is that IFS score metric itself may not capture all relevant aspects of student productivity. We used IFS despite its possible over-simplification because it has been shown to be a strong predictor of success in academic job markets [11]. Further, total impact factor score has been shown to be more predictive of fellowship application success than measures based only on first-author publications or number of citations [5]. Thus, for the purposes of illustrating the theoretical argument, we favored the IFS metric over h-index [12–14] because of its simplicity and similarity to previously validated approaches. Indeed, it might be argued that IFS shows more sensitivity and range of scores for young graduates and current students, as their (likely small number of) publications will largely be recent and therefore have been cited few times. Similarly, although we relied on a single measure of university rank via *U*.*S*. *News and World Report* rankings to define university tier, the validity of our theoretical argument does not rest on the particularities of the decision to rely on *U*.*S*. *News and World Report* rankings or the choice of IFS as our meritocratic measure.

Another possible concern is that our IFS metric does not capture all the possible factors contributing to faculty hiring decisions. For example, it could be suggested that IFS represents only one element that would contribute to an overall subjective assessment of a candidate’s productivity or merit; other factors might include conference presentations, oral presentation skills, teaching abilities, etc. Indeed, the ultimate decision to hire a candidate will of course be a function of a number of these factors, which can reasonably be assumed to aggregate into an overall subjective assessment. However, while certainly any comprehensive generative model of the faculty hiring process would necessitate both measuring and validating exactly what weighted combination of these and other possible factors would produce such a subjective assessment, such an undertaking is beyond the scope of this project. Our intent here was to provide simple demonstration that a six-fold difference in faculty hiring rates need not *necessitate* a six-fold difference in student productivity, and future studies should explore the construction of the full generative model.

The assumption of our simplified example that graduates seeking faculty positions who are not hired do not cumulatively add to the faculty applicant pool from year to year is certainly unrealistic. It is quite likely that at least some of the remaining 95% of graduate students who are not hired would be added to the following years’ new applicants, while some of them would take up a post-doctoral or a non-tenure track position before getting a tenure-track job. Nonetheless, there are reasons to expect a long transition period and the existing hierarchy between tenure-track and non-tenure track positions could result in an even more extreme criterion, leading to even more inequality in job placement since we expect cumulative advantage [18–20] to exacerbate the differences in IFS between Higher and Lower tier universities during the pre-tenure track years. Even if the post-doctoral job market is not as competitive as the faculty job market and thus has an equalizing effect, we are skeptical that the effect is enough to compensate for the impact of the current high threshold in the faculty job market.

Despite these potential criticisms of the methods we used to collect and define our proof-of-concept dataset, the predictions of the signal detection theoretic model do not depend on these choices. The signal detection theoretic model—demonstrating that a small difference in meritocratic measures across university rank can lead to a manifold difference in hiring rates under an extreme criterion—holds in almost any distribution (save distributions that are unlikely in empirical environments, e.g. rectangular/uniform distributions), and so the sample we gathered serves primarily to provide a concrete example of our theoretical argument.

## Conclusions

We have shown that similarly productive cohorts will produce very different rates of faculty hires simply because of a high hiring threshold, by demonstrating the predictions of a theoretical model with a realistic dataset. It should be noted that our results *cannot* definitively speak to whether or not the current system is a in fact pure meritocracy (see e.g., [11] for discussion of the impact of university rank and gender on faculty hiring). However, our demonstration does make clear that a substantial discrepancy in hiring rates as a function of degree-granting university tier *may not*, in fact, *necessitate* factors beyond the meritocratic.

## Supporting information

S1 File Raw data. This spreadsheet file contains the raw data for the analyses presented here.
(XLSX)
